# Association of coronary artery calcium and carotid intima-media thickness with white matter hyperintensity

**DOI:** 10.1097/MD.0000000000048823

**Published:** 2026-05-08

**Authors:** Seunghyeon Shin

**Affiliations:** a Department of Nuclear Medicine, Samsung Changwon Hospital, Sungkyunkwan University School of Medicine, Changwon, Republic of Korea.

**Keywords:** atherosclerosis, carotid intima-media thickness, coronary artery calcium score, risk factor, white matter hyperintensity

## Abstract

This study aimed to investigate the associations of coronary artery calcium (CAC) and carotid intima-media thickness (CIMT) with white matter hyperintensity (WMH). A total of 155 participants who underwent computed tomography for CAC scoring, carotid ultrasound, and brain magnetic resonance imaging within 2 days were included in this study. Univariate logistic analysis was performed to identify the variables to include in multivariate analysis. Age, sex, hypertension, diabetes mellitus, dyslipidemia, smoking history, body mass index, systolic blood pressure, diastolic blood pressure, fasting blood glucose, HbA1C, total cholesterol, triglyceride, high-density lipoprotein cholesterol, and low-density lipoprotein cholesterol levels were included as variables. After univariate logistic analysis, the variables which showed significance (*P* < .05) were included in multivariate logistic analysis for each CAC, CIMT, and WMH. To identify the effect of CAC and CIMT on WMH, multivariate logistic regression analysis of WMH also included the status of CAC and CIMT. In the multivariate logistic regression analysis, age (odds ratio [OR] 1.0747; confidence interval [CI] 1.0223–1.1279), female sex (OR 0.2924; CI 0.0875–0.9770), and hypertension (OR 2.3666; CI 1.0570–5.2990) were associated with CAC. Age (OR 1.1724; CI 1.1003–1.2493) and diabetes mellitus (OR 3.1934; CI 1.1236–9.0760) were associated with CIMT. Age (OR 1.1287; CI 1.0605–1.2014) and CAC positive (OR 2.3087; CI 1.0630–5.0145) were associated with WMH. CAC, but not CIMT, was significantly associated with WMH.

## 1. Introduction

Atherosclerosis is a chronic inflammatory disease characterized by endothelial dysfunction, intimal thickening, inflammation, and vascular calcification.^[1,2]^ Atherosclerotic intimal calcification is usually associated with atherosclerosis progression^[3]^ and is the dominant type of calcification in the coronary artery.^[4]^ Coronary artery calcium (CAC) can be measured using computed tomography (CT), and is a reliable predictor of coronary heart disease.^[5]^ Age, sex, race, hypertension (HTN), obesity, diabetes mellitus (DM), hypercholesterolemia, and a family history of coronary heart disease are associated with CAC.^[6,7]^ Furthermore, age, sex, high-density lipoprotein cholesterol (HDL-C), and total cholesterol were significantly associated with CAC progression.^[8]^

Carotid intima-media thickness (CIMT) is a surrogate marker for atherosclerosis and is useful for cardiovascular risk assessment.^[9]^ A meta-analysis reported that CIMT is strongly associated with cardiovascular disease events.^[10]^ However, another meta-analysis reported that CIMT showed only a small improvement in risk assessment.^[11]^ The CIMT is associated with age, systolic blood pressure, fasting glucose, total cholesterol, HDL-C, low-density lipoprotein cholesterol (LDL-C), and waist circumference.^[12]^

White matter hyperintensities (WMHs) are areas of increased signal intensity on T2-weighted fluid-attenuated inversion recovery (FLAIR) magnetic resonance imaging (MRI).^[13]^ WMHs are a common finding among older adults.^[14]^ However, WMHs are associated with a risk of cognitive decline, dementia, mood disorders, and gait abnormalities.^[13]^ Moreover, WMH volume is associated with DM, HTN, stroke, current smoking, body mass index (BMI), higher alcohol intake, insufficient physical activity,^[15]^ and future cardiovascular disease risk.^[16]^

As mentioned above, CAC, CIMT, and WMH share common risk factors. Thus, many studies were conducted to evaluate the association among them. Previous studies on the association between CIMT and WMH have shown inconsistent results.^[17–21]^ However, the results of studies on the association between CAC and WMH appear to be more consistent.^[22,23]^ Studies that compared CAC and WMH within the same subjects are rare. Thus, this study aimed to investigate the associations of CAC and CIMT with WMH.

## 2. Methods

### 2.1. Participants

**Figure 1.**
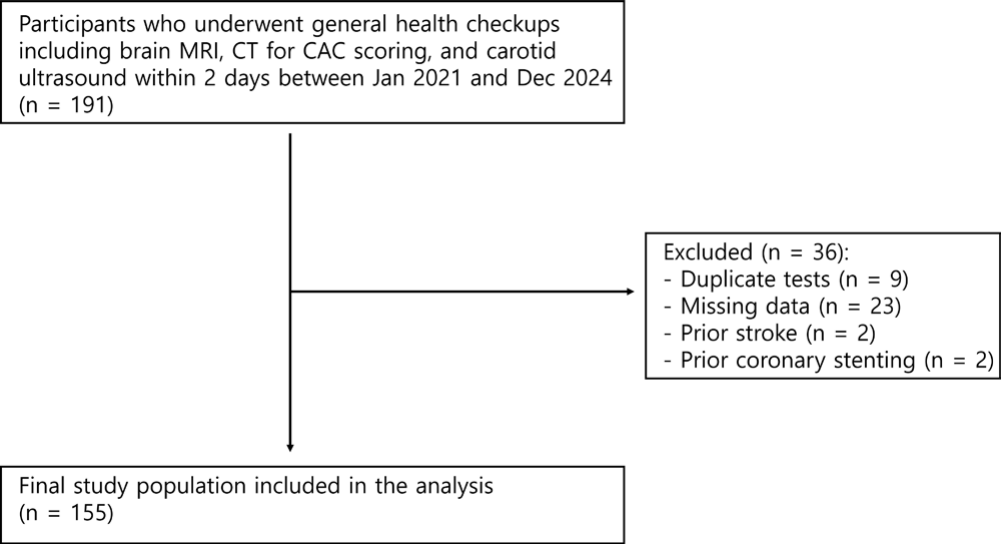
Flow diagram of study participant selection.

Data of participants who underwent general health checkups at Samsung Changwon Hospital between January 2021 and December 2024 were retrospectively reviewed. We identified the participants who underwent brain MRI, CT for CAC scoring, and carotid US within 2 days and a total of 191 participants met these criteria. After excluding participants with duplicate tests during the study period (n = 9), missing data (n = 23), prior stroke (n = 2), and prior coronary stenting (n = 2), a total of 155 participants with complete data were included in the final analysis (Fig. 1).

Data were obtained regarding age, sex, height, weight, systolic blood pressure (BP), diastolic BP, history of HTN, DM, dyslipidemia, coronary artery disease, stroke, smoking history, BMI (kg/m^2^), fasting blood glucose (mg/dL), glycated hemoglobin (HbA1c, %), total cholesterol (mg/dL), HDL-C (mg/dL), LDL-C (mg/dL), and triglyceride (mg/dL) levels. All laboratory tests were performed after overnight fasting. HTN was defined as self-reported hypertension, self-reported use of antihypertensive medication, or blood pressure ≥140/90 mm Hg. DM was defined as self-reported DM, self-reported use of insulin or hypoglycemic medication, fasting blood glucose ≥126 mg/dL, or HbA1c ≥6.5 %. Dyslipidemia was defined as self-reported dyslipidemia, self-reported use of lipid-lowering agent, or total cholesterol ≥240 mg/dL, LDL-C ≥160 mg/dL, HDL-C <40 mg/dL, or triglycerides ≥200 mg/dL. This study was approved by the Institutional Review Board of Samsung Changwon Hospital. The requirement for informed consent was waived because of the retrospective study design.

### 2.2. CT for CAC

CAC was measured using images acquired with a 64-section multidetector CT scanner (SOMATOM Definition AS, Siemens, Munich, Germany) in the non-contrast-enhanced mode. The subject laid down on an electrocardiogram. A CT scan was performed at the end of inspiration when the heart rate was <80 beats/min. The scan range was between the carina and cardiac apex. The scan parameters were as follows: 120 kVp, 70 mAs, and 3 mm slice thickness. The CT images were exported to an analysis software (Aquarius Ver.4.4.1 TeraRecon), which automatically produced the Agatston score. Lesions with 2 or more consecutive pixels and attenuation of >130 Hounsfield unit were defined as calcifications.

### 2.3. Carotid ultrasound

CIMT was measured by a trained sonographer according to standard procedures using high-resolution ultrasound (Vivid E90; General Electric, Vingmed, Milwaukee). CIMT measurements were performed with high-resolution B-mode ultrasonography of the common carotid artery (CCA) using a semiautomated edge detection software. A region of interest was positioned at the thickest area of the CCA after interrogation, and the mean of the average values of the left and right CCA was used for CIMT. The CIMT values between 0.6 and 0.7 mm are considered normal in healthy middle-aged adults.^[24]^ However, the American Society of Echocardiography reported that CIMT values greater than or equal to the 75th percentile for the patient’s age, sex, and race/ethnicity are considered high and indicative of increased CVD risk.^[25]^ In the context of the Korean population, CIMT values vary across studies. Cho et al reported mean CIMT values of 0.72 mm (male) and 0.67 mm (female) in healthy subjects aged 50 to 59 years.^[26]^ Conversely, a multicenter study by Bae et al observed lower mean values in healthy subjects aged 45 to 54 (0.61–0.65 mm), whereas patients with HTN and hyperlipidemia exhibited significantly higher mean values (0.69–0.73 mm).^[27]^ Furthermore, Youn et al demonstrated that the 75th percentile of mean CIMT in healthy subjects aged 50 to 59 years was 0.62 mm for males and 0.60 mm for females.^[28]^ As a value of 0.7 mm exceeds the 75th percentile for healthy subjects and aligns with the mean values observed in high-risk groups, the cutoff value of the abnormal CIMT was defined as 0.7 mm.

### 2.4. Brain MRI

T1-weighted and T2-weighted FLAIR sequences were used to measure white matter hyperintensities. Both images were acquired using Philips Ingenia 3T scanner (T1: TR = 479.5–500 ms and TE = 6.5–9.3 ms, T2: TR = 9000–10,000 ms and TE = 125 ms), Philips Ingenia CX 3T scanner (T1: TR = 450–480 ms and TE = 10.3–10.8 ms, T2: TR = 9000–11,000 ms and TE = 125 ms) (Philips Healthcare, Best, the Netherlands) and Siemens Skyra 3T scanner (T1: TR = 2000–2220 ms and TE = 27 ms, T2: TR = 9000 ms and TE = 85–94 ms) (Siemens Medical Solution, Erlangen, Germany).

All T1-weighted and FLAIR images were converted into a NIFTY file format using MRIcron. Lesions were segmented using the lesion growth algorithm^[29]^ as implemented in the LST toolbox (version 3.0.0; https://www.applied-statistics.de/lst.html) for statistical parametric mapping on MATLAB R2023b. The algorithm first segments the T1-images into 3 main tissue classes (cerebrospinal fluid, gray matter, and white matter). This information was then combined with the coregistered FLAIR intensities to calculate the lesion belief maps. By thresholding these maps with a pre-chosen initial threshold (κ = 0.3) an initial binary lesion map was obtained which was subsequently grown along voxels that appear hyperintense in the FLAIR image. The results were presented as a lesion probability map. WMH positive was defined as WMH volume >0 mL after application of the κ = 0.3 threshold. No additional manual minimum lesion size criterion was applied.

### 2.5. Statistical analysis

Descriptive analysis was performed to calculate the mean and standard deviation of the total sample. An independent *t* test was used to compare continuous variables between the positive and negative WMH groups, whereas a Chi-square test was used to compare categorical variables. Univariate logistic analysis was performed to identify the variables to include in multivariate analysis. Age, sex, HTN, DM, dyslipidemia, smoking history, BMI, systolic BP, diastolic BP, fasting blood glucose, HbA1C, total cholesterol, triglyceride, HDL-C, and LDL-C levels were included as variables. After univariate logistic analysis, the variables which showed significance (*P* < .05) were included in multivariate logistic analysis for each CAC, CIMT, and WMH. To identify the effect of CAC and CIMT on WMH, multivariate logistic regression analysis of WMH also included the status of CAC and CIMT. Multicollinearity was assessed by variance inflation factor. The Hosmer–Lemeshow test was performed to evaluate the goodness-of-fit of the logistic regression model. Two-sided *P*-values <.05 were considered significant. MedCalc Statistical Software version 23.2.1 (MedCalc Software Ltd, Ostend, Belgium; https://www.medcalc.org; 2025) was used for performing all the statistical analyses.

## 3. Results

### 3.1. Subject characteristics

**Figure 2.**
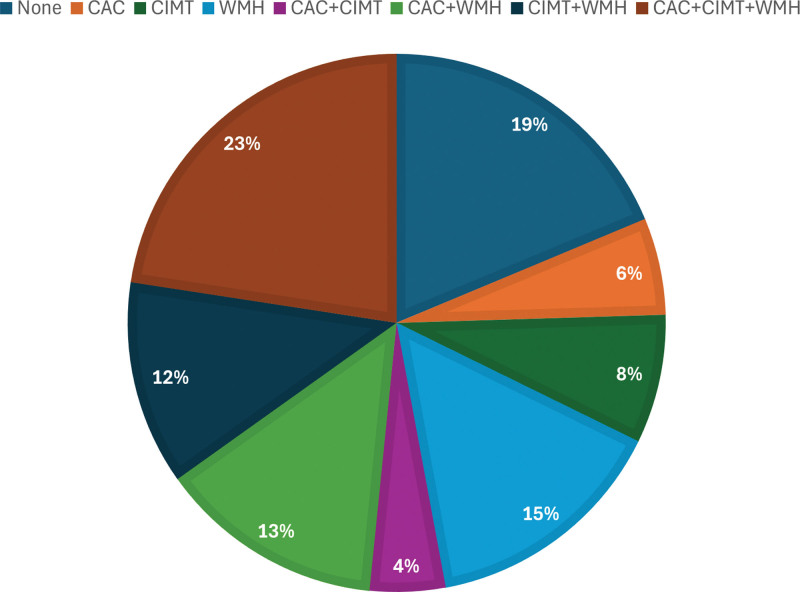
The distribution of CAC, CIMT, and WMH. CAC = coronary artery calcium, CIMT = carotid intima-media thickness, WMH = white matter hyperintensity.

A total of 155 participants (male-to-female ratio, 6:1) were included in this study. Mean age and BMI were 54.7 ± 8.1 years and 24.6 ± 3.1 kg/m^2^, respectively. A total of 72 patients had CAC, 73 subjects had abnormal CIMT, and 98 subjects had WMH. Twenty-nine patients had no CAC, abnormal CIMT, or WMH. Forty-four subjects had 1 abnormal result in the CAC, CIMT, and WMH groups (CAC, 9; CIMT, 12; WMH, 23). Seven patients had CAC and abnormal CIMT without WMH. Twenty-one patients had CAC and WMH without CIMT. Nineteen subjects had abnormal CIMT and WMH without CAC. Thirty-five subjects had CAC, abnormal CIMT, and WMH (Fig. 2). Detailed characteristics are shown in Table 1. When the subjects were divided into 2 groups according to WMH status, subjects with WMH were older, had more DM, higher fasting glucose, higher HbA1c, and higher CIMT than those without WMH (Table 2).

**Table 1:** Subjects’ characteristics.

	Subject (n = 155)
Age (years)	54.7 ± 8.1
Sex	
Male	133
Female	22
Hypertension	45
Diabetes mellitus	26
Dyslipidemia	65
Smoking	
Never smoking	75
Ex-smoking	51
Current smoking	29
Body mass index (kg/m^2^)	24.6 ± 3.1
Blood pressure	
Systolic (mm Hg)	120.6 ± 10.9
Diastolic (mm Hg)	74.5 ± 9.1
Fasting glucose (mg/dL)	103.7 ± 26.8
Total cholesterol (mg/dL)	194.5 ± 42.9
Triglyceride (mg/dL)	136.4 ± 93.5
HDL-C (mg/dL)	58.5 ± 14.9
LDL-C (mg/dL)	121.5 ± 40.0
HbA1c (%)	5.80 ± 1.03
CAC score	80.4 ± 249.1
CIMT (mm)	0.72 ± 0.17
WMH volume (mL)	0.27 ± 0.71
CAC positive/negative	72/83
CIMT abnormal/normal	73/82
WMH positive/negative	98/57

CAC = coronary artery calcium, CIMT = carotid intima-media thickness, HbA1c = glycated hemoglobin, HDL-C = high-density lipoprotein cholesterol, LDL-C = low-density lipoprotein cholesterol, WMH = white matter hyperintensity.

**Table 2:** Difference between negative and positive white matter hyperintensity.

	WMH negative (n = 57)	WMH positive (n = 98)	*P*-value
Age (yr)	50.5 ± 7.7	57.2 ± 7.3	<.0001
Sex			.6652
Male	48	85	
Female	9	13	
Hypertension (yes/no)	12/45	33/65	.0962
Diabetes mellitus (yes/no)	4/53	22/76	.0135
Dyslipidemia (yes/no)	25/32	40/58	.7121
Smoking			.8647
Never smoking	26	49	
Ex-smoking	20	31	
Current smoking	11	18	
Body mass index (kg/m^2^)	25.0 ± 3.8	24.5 ± 2.6	.3799
Blood pressure			
Systolic (mm Hg)	121.1 ± 10.5	120.2 ± 11.2	.6332
Diastolic (mm Hg)	75.7 ± 8.9	73.8 ± 9.2	.2025
Fasting glucose (mg/dL)	98.8 ± 10.7	106.5 ± 32.5	.0310
Total cholesterol (mg/dL)	196.7 ± 42.4	193.2 ± 43.4	.6308
Triglyceride (mg/dL)	140.5 ± 99.4	134.0 ± 90.4	.6788
HDL-C (mg/dL)	58.5 ± 14.1	58.5 ± 15.4	.9841
LDL-C (mg/dL)	123.9 ± 41.0	120.1 ± 39.4	.5749
HbA1c (%)	5.62 ± 0.37	5.91 ± 1.25	.0357
CAC score	39.2 ± 153.5	104.4 ± 288.6	.0684
CIMT (mm)	0.68 ± 0.18	0.74 ± 0.16	.0333
CAC positive/negative	16/41	56/42	.0005
CIMT abnormal/normal	19/38	54/44	.0091

CAC = coronary artery calcium, CIMT = carotid intima-media thickness, HbA1c = glycated hemoglobin, HDL-C = high-density lipoprotein cholesterol, LDL-C = low-density lipoprotein cholesterol, WMH = white matter hyperintensity.

### 3.2. Association between risk factors and each CAC, CIMT, and WMH

In the multivariate logistic regression analysis, age (odds ratio [OR] 1.0747; confidence interval [CI] 1.0223–1.1279), female sex (OR 0.2924; CI 0.0875–0.9770), and HTN (OR 2.3666; CI 1.0570–5.2990) were associated with CAC (Table 3). Age (OR 1.1724; CI 1.1003–1.2493) and DM (OR 3.1934; CI 1.1236–9.0760) were associated with CIMT (Table 4). Age (OR 1.1287; CI 1.0605–1.2014) and CAC positive (OR 2.3087; CI 1.0630–5.0145) were associated with WMH (Table 5). All variance inflation factor values were <5, indicating no significant multicollinearity. The Hosmer–Lemeshow test indicated adequate model fit (*P* > .05).

**Table 3:** Association between risk factors and CAC.

	Univariate			Multivariate		
	OR	95% CI	*P*-value	OR	95% CI	*P*-value
Age	1.0713	1.0249–1.1197	.0023	1.0747	1.0223–1.1297	.0047
Female	0.2124	0.0682–0.6612	.0075	0.2924	0.0875–0.9770	.0457
HTN	2.8241	1.3738–5.8054	.0047	2.3666	1.0570–5.2990	.0362
DM	3.8922	1.5282–9.9131	.0044	0.8147	0.1929–3.4403	.7804
Dyslipidemia	1.0897	0.5748–2.0656	.7924			
Past smoking	1.4750	0.7526–2.8909	.2576			
Current smoking	0.7765	0.3428–1.7586	.5441			
BMI	1.0278	0.9274–1.1391	.6006			
SBP	1.0129	0.9836–1.0430	.3915			
DBP	0.9946	0.9604–1.0300	.7600			
Fasting glucose	1.0335	1.0084–1.0592	.0087	1.0023	0.9679–1.0379	.8989
Total cholesterol	0.9974	0.9900–1.0048	.4850			
Triglyceride	0.9996	0.9962–1.0030	.8328			
HDL-C	0.9974	0.9763–1.0188	.8084			
LDL-C	0.9973	0.9894–1.0053	.5053			
HbA1c	3.9317	1.8159–8.5124	.0005	3.0176	0.9588–9.4972	.0590

BMI = body mass index, CI = confidence interval, DBP = diastolic blood pressure, DM = diabetes mellitus, HbA1c = glycated hemoglobin, HDL-C = high-density lipoprotein cholesterol, HTN = hypertension, LDL-C = low-density lipoprotein cholesterol, OR = odds ratio, SBP = systolic blood pressure.

**Table 4:** Association between risk factors and CIMT.

	Univariate			Multivariate		
	OR	95% CI	*P*-value	OR	95% CI	*P*-value
Age	1.1760	1.1049–1.2517	<.0001	1.1724	1.1003–1.2493	<.0001
Female	0.4737	0.1815–1.2365	.1269			
HTN	1.4227	0.7094–2.8535	.3207			
DM	3.7698	1.4807–9.5981	.0054	3.1934	1.1236–9.0760	.0294
Dyslipidemia	1.5965	0.8397–3.0352	.1535			
Past smoking	1.2613	0.6446–2.4679	.4979			
Current smoking	1.4899	0.6617–3.3544	.3356			
BMI	0.9790	0.8833–1.0851	.6866			
SBP	0.9964	0.9678–1.0258	.8070			
DBP	0.9706	0.9360–1.0065	.1069			
Fasting glucose	1.0169	0.9977–1.0364	.0849			
Total cholesterol	0.9983	0.9909–1.0057	.6457			
Triglyceride	0.9981	0.9945–1.0017	.3002			
HDL-C	0.9925	0.9714–1.0140	.4903			
LDL-C	1.0002	0.9923–1.0081	.9631			
HbA1c	1.7175	0.9608–3.0702	.0680			

BMI = body mass index, CI = confidence interval, DBP = diastolic blood pressure, DM = diabetes mellitus, HbA1c = glycated hemoglobin, HDL-C = high-density lipoprotein cholesterol, HTN = hypertension, LDL-C = low-density lipoprotein cholesterol, OR = odds ratio, SBP = systolic blood pressure.

**Table 5:** Association between risk factors and WMH.

	Univariate			Multivariate		
	OR	95% CI	*P*-value	OR	95% CI	*P*-value
Age	1.1397	1.0773–1.2057	<.0001	1.1287	1.0605–1.2014	.0001
Female	0.8157	0.3249–2.0481	.6645			
HTN	1.9038	0.8884–4.0801	.0978			
DM	3.8355	1.2494–11.7745	.0188	2.4922	0.7272–8.5413	.1462
Dyslipidemia	0.8828	0.4561–1.7085	.7113			
Past smoking	0.8560	0.4290–1.7079	.6590			
Current smoking	0.9409	0.4090–2.1647	.8861			
BMI	0.9487	0.8532–1.0549	.3308			
SBP	0.9927	0.9634–1.0229	.6310			
DBP	0.9767	0.9418–1.0129	.2035			
Fasting glucose	1.0209	0.9970–1.0453	.0866			
Total cholesterol	0.9981	0.9905–1.0058	.6284			
Triglyceride	0.9993	0.9958–1.0027	.6773			
HDL-C	0.9998	0.9781–1.0220	.9840			
LDL-C	0.9976	0.9895–1.0058	.5724			
HbA1c	1.8783	0.9597–3.6761	.0658			
CAC positive	3.4167	1.6920–6.8993	.0006	2.3087	1.0630–5.0145	.0345
CIMT abnormal	2.4545	1.2441–4.8426	.0096	0.9046	0.3976–2.0582	.8110

BMI = body mass index, CAC = coronary artery calcium, CI = confidence interval, CIMT = carotid intima-media thickness, DBP = diastolic blood pressure, DM = diabetes mellitus, HbA1c = glycated hemoglobin, HDL-C = high-density lipoprotein cholesterol, HTN = hypertension, LDL-C = low-density lipoprotein cholesterol, OR = odds ratio, SBP = systolic blood pressure, WMH = white matter hyperintensity.

## 4. Discussion

In this study, we demonstrated that age, female sex, and HTN were independently associated with CAC. Age and DM were independently associated with CIMT. Age and CAC positive were independently associated with WMH.

Vascular calcification is the deposition of calcium-phosphate complex in vessel wall.^[30,31]^ Vascular calcification is divided into 2 types depending on its location and etiology.^[30,31]^ One is atherosclerotic intimal calcification, which is affected by lipid deposition, inflammation, and necrosis^[30,31]^ and the other is medial calcification, which occurs in the medial layer of the vessel wall and is often associated with chronic kidney disease, DM, hypertension, osteoporosis, and aging.^[30,31]^ Vascular calcification, either intimal calcification or medial calcification, is associated with aging.^[30,32]^ However, intimal calcification is the dominant type of calcification in coronary artery.^[4]^ CAC is a feature of advanced atherosclerosis and is known to be a predictor of future cardiac events.^[4]^ Previous studies reported that age was associated with CAC and prevalence of CAC was higher in males than females, consistent with this study.^[33,34]^ In addition, HTN is an independent predictors of CAC, which is consistent with the results of this study.^[34]^

The CIMT is associated with age.^[35]^ Age-related vascular remodeling leads to vascular wall thickening through alterations in both the intima and media.^[36]^ The underlying mechanisms include vascular smooth muscle cell proliferation and migration, extracellular matrix deposition, and impaired elastin integrity.^[36]^ In the carotid artery, intimal thickness increases with aging, accompanied by a progressive increase in medial thickness.^[36]^ Intimal thickening primarily results from vascular smooth muscle cell migration and accumulation of subendothelial extracellular matrix components, including collagen and proteoglycans.^[36]^ Medial thickening is mainly driven by smooth muscle cell proliferation and increased collagen content.^[36]^ In the aging carotid artery, the media is characterized by reduced elastin content and increased collagen.^[36]^ This underlying mechanism may explain the association between the CIMT and age. A previous study reported that CIMT in DM subjects was higher than that in non-DM subjects, consistent with this study.^[37]^

WMH is known to increase with age.^[38]^ WMH is the most common feature of small vessel disease (SVD) on MRI.^[38]^ SVD is a chronic progressive disorder affecting small vessels which are typically 50 to 400 um in diameter.^[39]^ One of the most common SVD pathologies is arteriolosclerosis caused by aging, hypertension, and other conventional vascular risk factors.^[40]^

In the present study, CAC was associated with WMH, whereas CIMT was not significantly associated with WMH. Previous studies have reported a significant association between CIMT and WMH in hypertensive patients and midlife women.^[17,18]^ However, other studies demonstrated no significant association.^[19–21]^ Notably, previous studies showing no association had a relatively lower mean age than those showing a significant association. The number of enrolled subjects in the studies showing no association was higher than that of enrolled subjects in the studies showing a significant association. Della-Morte et al study demonstrated that CIMT was associated with WMH in participants 70 years or older.^[41]^ These results suggest that the underlying mechanisms of CIMT and WMH differ. According to a statement from the American Society of Echocardiography, CIMT is a feature of arterial wall aging rather than subclinical atherosclerosis.^[25]^ However, CIMT is known to relate to atherosclerosis as the underlying mechanism of CIMT is implicated in the development and progression of atherosclerosis.^[25]^ This may explain the inconsistent association between CIMT and WMH according to age.

Consistent with this study, previous studies have reported that CAC was associated with WMH in middle-aged and elderly adults and predicted WMH progression in elderly adults.^[22,23,42]^ Arteriolosclerosis, a common feature of SVD presented by WMH on MRI, is the thickening and hardening of the arteriolar walls.^[1]^ Increased arterial stiffness is a possible early marker of arteriolosclerosis.^[43]^ Arterial stiffness measured using pulse wave velocity is associated with WMH^[44]^ and its progression.^[45]^ In addition, increased arterial stiffness is associated with calcium levels in the coronary arteries.^[46–48]^ These results suggest a connection between CAC and WMH. Devantier et al evaluated the association between coronary plaque volume, CIMT, and WMH and reported inconsistent results.^[49]^ They reported that WMH was associated with CIMT, but not with coronary plaque volume.^[49]^ However, the enrolled number of the study of Devantier et al is smaller than that of this study (56 vs 155), and they investigated coronary plaque volume, which consisted of calcified and noncalcified plaque volumes. These differences may have contributed to these inconsistent results.

This study had several limitations. First, owing to the retrospective design of the study, selection bias was inevitable. Second, the enrolled number of the study is relatively small. Third, only middle-aged adults were included in this study. However, this association may differ according to age. Fourth, MRI data used in this study were derived from various scanners and protocols. This variability could influence WMH detection and quantification. Fifth, although medication use was included in the definitions of HTN, DM, and dyslipidemia, the potential independent effects of specific medications on CAC and WMH could not be fully assessed, which may have resulted in residual confounding.

In conclusion, age, female sex, and HTN were independently associated with CAC. Age and DM were independently associated with CIMT. Age and CAC positive were independently associated with WMH. CAC, but not CIMT, was significantly associated with WMH.

## Author contributions

**Conceptualization:** Seunghyeon Shin.

**Data curation:** Seunghyeon Shin.

**Formal analysis:** Seunghyeon Shin.

**Funding acquisition:** Seunghyeon Shin.

**Investigation:** Seunghyeon Shin.

**Methodology:** Seunghyeon Shin.

**Project administration:** Seunghyeon Shin.

**Resources:** Seunghyeon Shin.

**Software:** Seunghyeon Shin.

**Supervision:** Seunghyeon Shin.

**Validation:** Seunghyeon Shin.

**Visualization:** Seunghyeon Shin.

**Writing – original draft:** Seunghyeon Shin.

**Writing – review & editing:** Seunghyeon Shin.
